# Peatland vegetation composition and phenology drive the seasonal trajectory of maximum gross primary production

**DOI:** 10.1038/s41598-018-26147-4

**Published:** 2018-05-22

**Authors:** Matthias Peichl, Michal Gažovič, Ilse Vermeij, Eefje de Goede, Oliver Sonnentag, Juul Limpens, Mats B. Nilsson

**Affiliations:** 1 Department of Forest Ecology and Management, Swedish University of Agricultural Sciences, 90183 Umeå, Sweden; 2 Plant Ecology and Nature Conservation Group, Wageningen University, 6708 PB Wageningen, The Netherlands; 3 Department of Aquatic Ecology, Radboud University Nijmegen, 6525 AJ Nijmegen, The Netherlands; 4 Institute of Environmental Sciences, Leiden University, 2333CC Leiden, The Netherlands; 5 Département de géographie, Université de Montréal, Montréal, QC H2V 2B8 Canada

## Abstract

Gross primary production (GPP) is a key driver of the peatland carbon cycle. Although many studies have explored the apparent GPP under natural light conditions, knowledge of the maximum GPP at light-saturation (GPP_max_) and its spatio-temporal variation is limited. This information, however, is crucial since GPP_max_ essentially constrains the upper boundary for apparent GPP. Using chamber measurements combined with an external light source across experimental plots where vegetation composition was altered through long-term (20-year) nitrogen addition and artificial warming, we could quantify GPP_max_ *in-situ* and disentangle its biotic and abiotic controls in a boreal peatland. We found large spatial and temporal variations in the magnitudes of GPP_max_ which were related to vegetation species composition and phenology rather than abiotic factors. Specifically, we identified vegetation phenology as the main driver of the seasonal GPP_max_ trajectory. Abiotic anomalies (i.e. in air temperature and water table level), however, caused species-specific divergence between the trajectories of GPP_max_ and plant development. Our study demonstrates that photosynthetically active biomass constrains the potential peatland photosynthesis while abiotic factors act as secondary modifiers. This further calls for a better representation of species-specific vegetation phenology in process-based peatland models to improve predictions of global change impacts on the peatland carbon cycle.

## Introduction

Northern peatlands play a crucial role in the global carbon cycle storing almost one third of the global soil carbon pool^1–3^ and providing a small but persistent sink for atmospheric carbon dioxide (CO_2_)^4,5^. During recent decades, however, the continued peatland carbon sink strength has been questioned due to projected changes in global climate and increasing human pressure^6–8^. This concern is primarily based on studies proposing increased peat mineralization under warmer and drier conditions^9–11^. Meanwhile, the concurrent impact from global changes on the peatland gross primary production (GPP) has frequently been overlooked in this debate. GPP is, however, a major driver of the peatland carbon cycle as it determines not only the amount of atmospheric CO_2_ sequestered into biomass but also fuels microbial decomposition as well as the production of methane and dissolved organic carbon^12–15^. Thus, detailed knowledge of spatio-temporal GPP dynamics and its controls is imperative for improving our predictions of peatland ecology, biogeochemistry and carbon balance in response to global change.

Light availability is a key control of GPP^16,17^. Due to the difficulty in controlling light levels *in-situ*, however, field studies commonly explore apparent GPP under varying natural light conditions. As a consequence, our understanding of maximum GPP (GPP_max_) at light-saturation level is currently limited. This knowledge gap is critical since GPP_max_ essentially constrains the seasonal variations of the apparent GPP. Conceptually, GPP_max_ at the ecosystem level is the sum of species-specific photosynthesis under non-limiting light conditions (*P*_*sat*_) and its main functional modifiers (*f*; ranging from 0 to 1) including photosynthetically active biomass (*B*), temperature (*T*) and water availability (*W*) integrated over the number of species (*n*) within a reference time frame (e.g. annual) (Eq. 1)^17,18^. Vapor pressure deficit (which scales with combined *T* and *W* dynamics) and long-term (i.e. decadal) changes in atmospheric CO_2_ concentration might further affect photosynthesis rates^19–21^.

1 $${{\rm{GPP}}}_{{\rm{\max }}}={\sum }_{{i}=1}^{{n}}{P}_{sat}\times f(B)\times f(T)\times f(W)$$

Previous studies have approximated GPP_max_ using the parameter describing GPP at the light-saturation level (i.e. A_max_) in light response functions developed from field data collected under variable natural light conditions^22–25^ or from controlled laboratory measurements^26,27^. Although GPP_max_ is in theory equivalent to A_max_ in representing a measure of photosynthesis at light saturation level, here, we assign a different notation to differentiate estimates of light-saturated GPP from *in-situ* measurements (i.e. GPP_max_) *versus* estimates derived from a fitted model parameter (i.e. A_max_). Furthermore, since non-limiting light conditions are a prerequisite for achieving potential GPP (where *f*(B, T, W) = 1, i.e. at optimum), improved knowledge of GPP_max_ may be considered a step towards a better understanding of the potential GPP.

Given its dependence on plant development, temperature and water availability (Eq. 1), variations in GPP_max_ may occur across spatial (within and across peatland types) and temporal (intra- and inter-annual) scales. Specifically, species-specific differences in photosynthetic capacity, growth and survival strategies^27–31^ as well as inter-species competition dynamics^32,33^ may alter GPP_max_ patterns at the plant-community level. These biotic effects might be further modified by concurrent constraints from temperature and water availability^34–36^. To date, the spatial and temporal patterns of peatland GPP_max_ in response to its individual biotic and abiotic controls are poorly understood. Such knowledge, however, is needed to advance our understanding of global change impacts on the peatland carbon cycle and to help improve GPP estimates based on remote sensing products^37–39^ and process-based ecosystem models^40–46^.

Separating the biotic and abiotic controls of carbon fluxes in peatland ecosystems is challenging since information on vegetation development is often compromised by coarse temporal and spatial resolution^38,47^. To overcome this limitation, phenology cameras (i.e. digital repeat photography) have been used to track changes in canopy greenness as a proxy for peatland vegetation development^48,49^. The greenness index extracted from digital images has the advantage of integrating both quantitative (i.e. leaf area) and physiological (i.e. leaf chlorophyll and carotenoid contents) information across various spatial scales (plot to ecosystem) at quasi continuous temporal resolution^37,50–53^ and offers therefore new opportunities for exploring vegetation phenology controls on the peatland GPP.

In this study, we used a unique experimental set-up that included chamber measurements in combination with an external light source to quantify GPP_max_ *in-situ* across an array of long-term (20-year) vegetation manipulation plots in a boreal peatland over three meteorologically different growing seasons. Specifically, measurements were carried out in a natural control plot (C), a moss plot (M) where vascular plants were manually removed, and in plots were nitrogen addition (N) and artificial warming combined with nitrogen addition (WN) have caused a gradual shift in vegetation composition (see Supplementary Fig. S1 and Methods section for further details). In addition, we collected comprehensive biotic data using phenology cameras, spectral reflectance measurements and vegetation inventories. Our main objectives were to i) determine the magnitude and seasonal trajectory of GPP_max_ across different vegetation communities and ii) separate the effects of biotic and abiotic variables on GPP_max_.

## Results

### Magnitudes and seasonal patterns of GPP_max_

**Figure 1:**
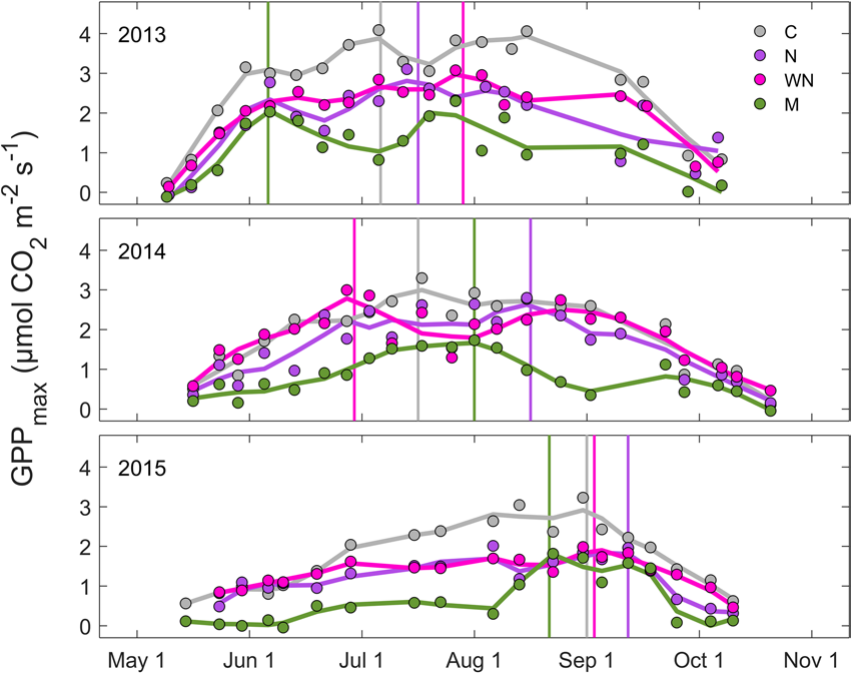
*In-situ* measurements of maximum gross primary production (GPP_max_) under full light conditions (2000 µmol m^−2^ s^−1^) for control (C), nitrogen (N), warming + N (WN) and moss (M) plots during the growing seasons of 2013–2015. Vertical lines indicate the timing of peak GPP_max_.

Our results show that the magnitudes and seasonal patterns of GPP_max_ differed considerably among years and plots (Fig. 1). In the C plot, mean GPP_max_ from June to August in 2013 and 2015 was significantly higher than in the other plots (Supplementary Fig. S2). Meanwhile, GPP_max_ was generally lowest in the M plot in all years, except for a short period in September 2015 when it matched GPP_max_ in the N and WN plots. GPP_max_ reached maximum values of 4.1, 3.1, 3.1 and 2.3 µmol CO_2_ m^−2^ s^−1^ in the C, N, WN and M plots, respectively.

The timing of peak GPP_max_ varied by several weeks among years and plots (Fig. 1). In some cases (e.g. for the C and M plots in 2013), a secondary peak period occurred within the same season. The earliest and latest occurrences were noted in 2013 and 2015, respectively, for all but the WN plot. In the WN plot, peak GPP_max_ occurred earlier in 2014 than in 2013, possibly due to the temporary decrease of GPP_max_ in late July 2014. During the same period, GPP_max_ was not reduced in the C and N plots and peaked in the M plot.

**Figure 2:**
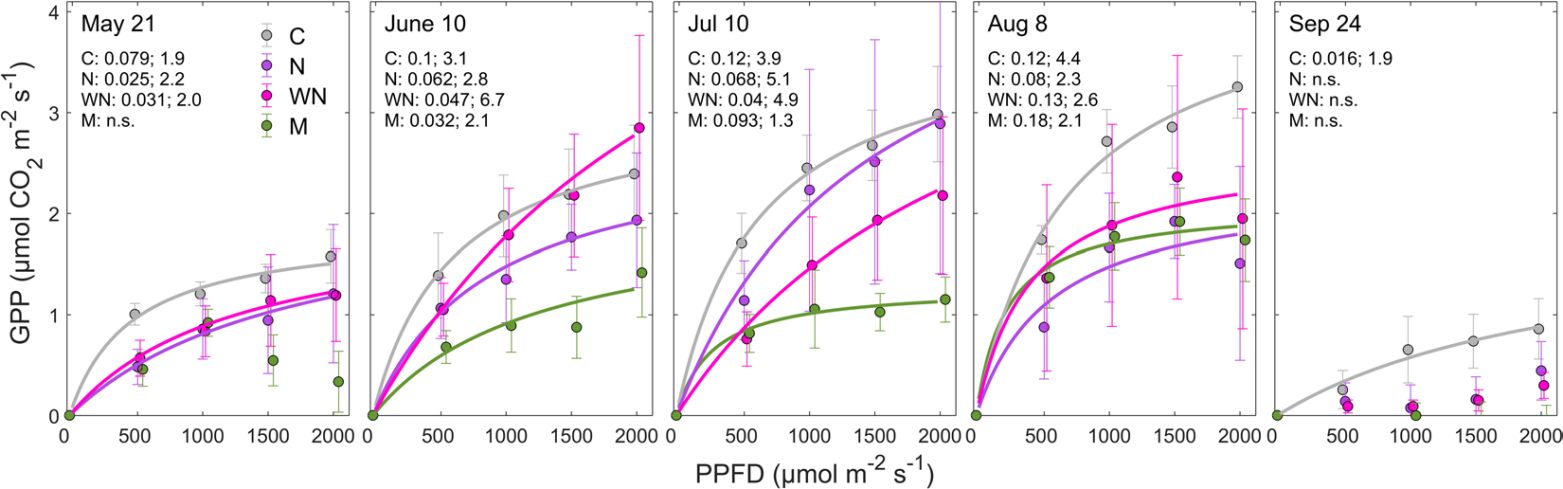
*In-situ* measurements of gross primary production (GPP) at photosynthetic photon flux density (PPFD) levels of 0, 500, 1000, 1500 and 2000 µmol m^−2^ s^−1^ for control (C), nitrogen (N), warming + N (WN) and moss (M) plots in 2013. Dots represent measured data; error bars indicate standard error; line shows hyperbolic fit when parameters α and A_max_ could be estimated. The first and second values following the plot acronym show α and A_max_ values from the hyperbolic fit, respectively; n.s. indicates non-significant fits (*p* > 0.05).

Temporal and spatial differences in the photosynthetic capacity were also evident in the GPP-light response parameters α and A_max,_ which varied considerably among months and plots during 2013 (Fig. 2). Specifically, in the WN and N plots (both dominated by vascular plants), A_max_ peaked in June and July, respectively, while its peak was delayed until August in the C and M plots (both with 100% moss cover). Among plots containing vascular plants, α was highest in the C plot in all months except for August when α in the C and WN plots were similar. In the M plot, α was lowest in June but thereafter increased and exceeded α in all other plots in August.

### Biotic and abiotic controls of the seasonal GPP_max_ trajectory

**Figure 3:**
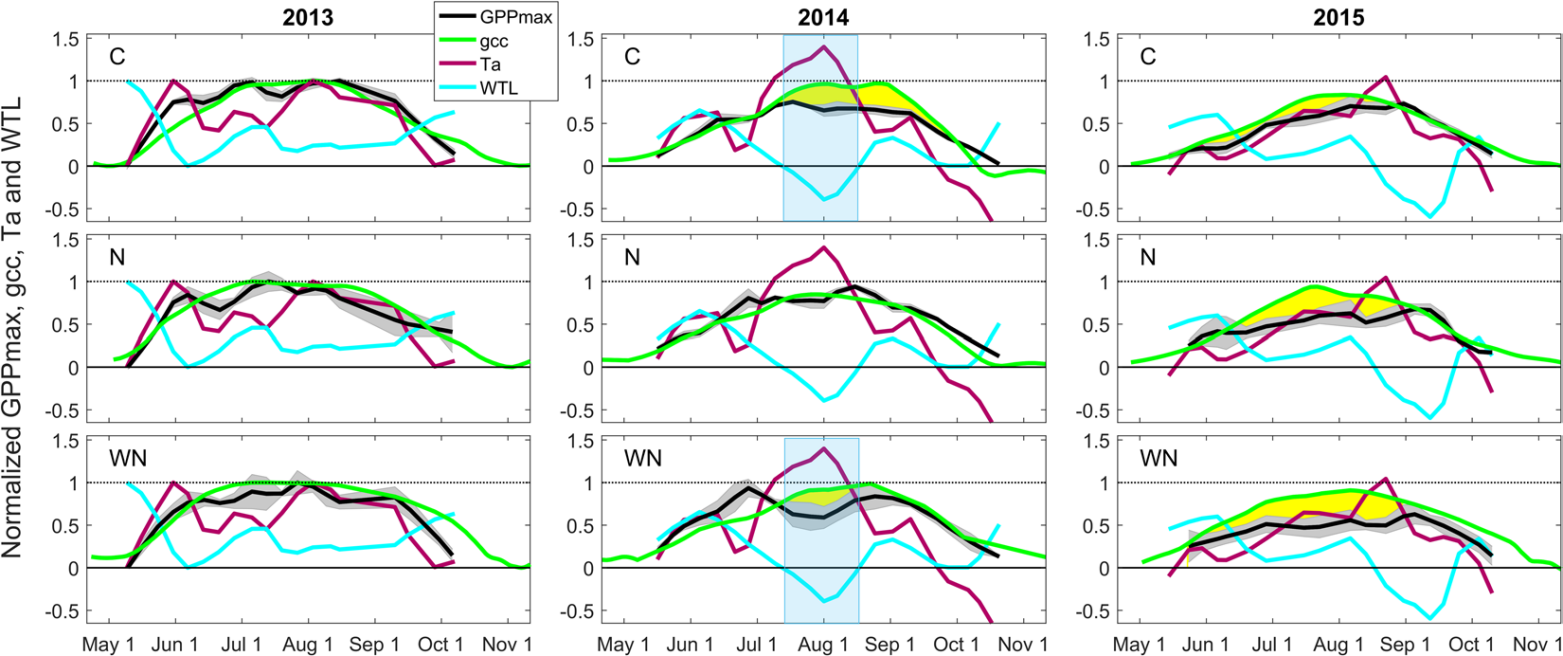
Normalized trajectory lines of maximum gross primary production (GPP_max_), canopy greenness index (gcc), air temperature (Ta) and water table level (WTL) in the control (C; top row), nitrogen (N; middle row) and warming + N (WN, bottom row) plots in 2013 (1^st^ column), 2014 (2^nd^ column) and 2015 (3^rd^ column). 2014 and 2015 trajectories were normalized relative to minimum and maximum (i.e. 0 and 1) of trajectory lines in 2013 which was considered a ‘meteorologically normal’ reference year. Grey shaded bands indicate standard error of the GPP_max_ trajectory. The yellow shaded areas visualize extended divergence between the trajectories of GPP_max_ and gcc; blue shaded boxes highlight a period of low WTL and high Ta corresponding to a divergence in GPP_max_ and gcc trajectories.

**Figure 4:**
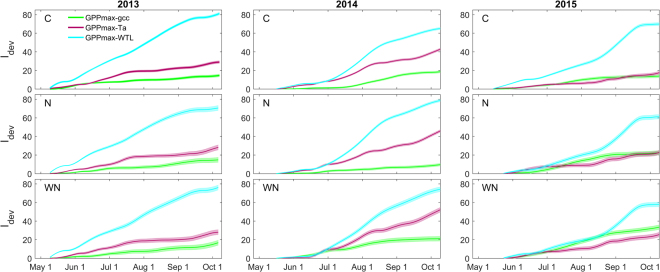
Cumulative sums of the deviation index (I_dev_; see Supplementary Fig. S7) describing the departure of the normalized trajectory line of maximum gross primary production (GPP_max_) from the trajectory lines of canopy greenness index (gcc), air temperature (Ta) and water table level (WTL) in the control (C; top row), nitrogen (N; middle row) and warming + N (WN, bottom row) plots in 2013 (1^st^ column), 2014 (2^nd^ column) and 2015 (3^rd^ column). Shaded bands indicate the standard error of the mean trajectory lines.

The seasonal trajectory of GPP_max_ was in general best explained (i.e. having the smallest cumulative deviation index, I_dev_) by the trajectory of gcc (i.e. plant phenology) rather than that of air temperature or water table level in the three plots containing vascular plants (Figs 3 and 4). One exception was the summer period in 2014 during which the trajectories of GPP_max_ and gcc temporarily deviated in the C and WN plots. In contrast, GPP_max_ and gcc trajectories did not diverge in the N plot during this period. Another exception occurred in 2015 when the trajectory of GPP_max_ deviated from the trajectory of gcc and instead followed most closely that of air temperature in all three plots.

### Vegetation biomass, species composition and phenology

**Figure 5:**
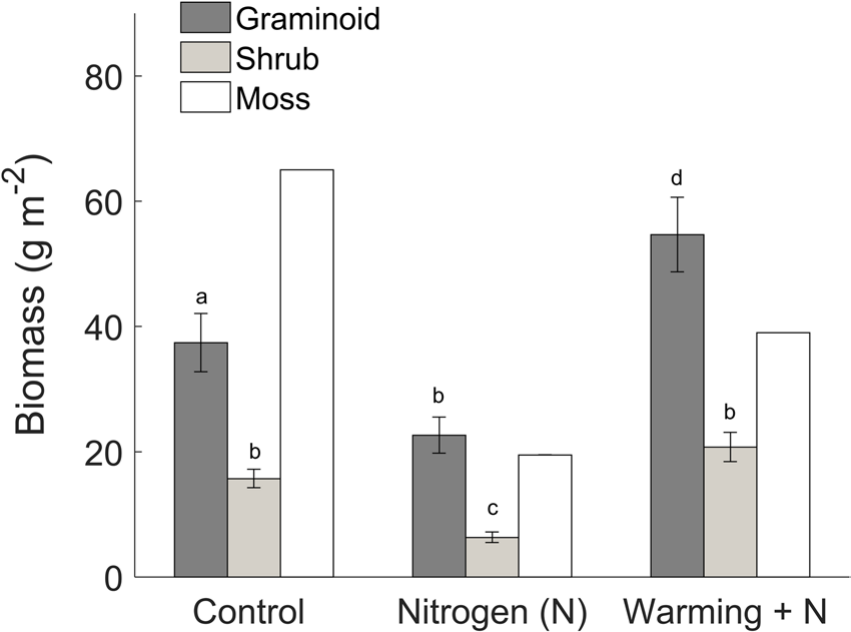
Mean (2013–2015) aboveground biomass of graminoids (*E*. *vaginatum* and *T*. *cespitosum*) and woody shrubs (*V*. *oxycoccos*, *A*. *polifolia* and *R*. *chamaemorus*) in control (C), nitrogen (N) and warming + N (WN) plots (*n* = 10); error bars indicate ± 2SE and different letters indicate significant (*p* < 0.05) differences among means across and within plots based on a non-parametric Friedman one-way analysis of variance by ranks test for repeated measurements followed by a Bonferroni post-hoc comparison; moss biomass (i.e. capitula) in the C plot was adopted from Laine *et al*.^63^ and adjusted by differences in moss area cover to obtain estimates for the N and WN plots. Since only one estimate for moss area was available at each plot (i.e. *n* = 1), moss biomass was excluded from the statistical analysis.

To explore the drivers for the observed variations in the magnitudes and trajectories of GPP_max_, it is necessary to take into account the considerable differences in aboveground biomass and relative species contribution among the plots (Fig. 5). Specifically, moss cover was 100, 30 and 58% in the C, N and WN plots, respectively, translating into similar differences in moss (capitulum) biomass. In the C plot, biomass was more evenly distributed across species groups with total vascular plant biomass being similar to moss biomass while in the N and WN plots vascular plant biomass was almost twice as high as moss biomass. In all plots, graminoid biomass was significantly greater than shrub biomass. In the N plot, both graminoid and shrub biomass were significantly lower than in the C and WN plots. Graminoid biomass in the WN plot was significantly higher than in the C and N plots.

**Figure 6:**
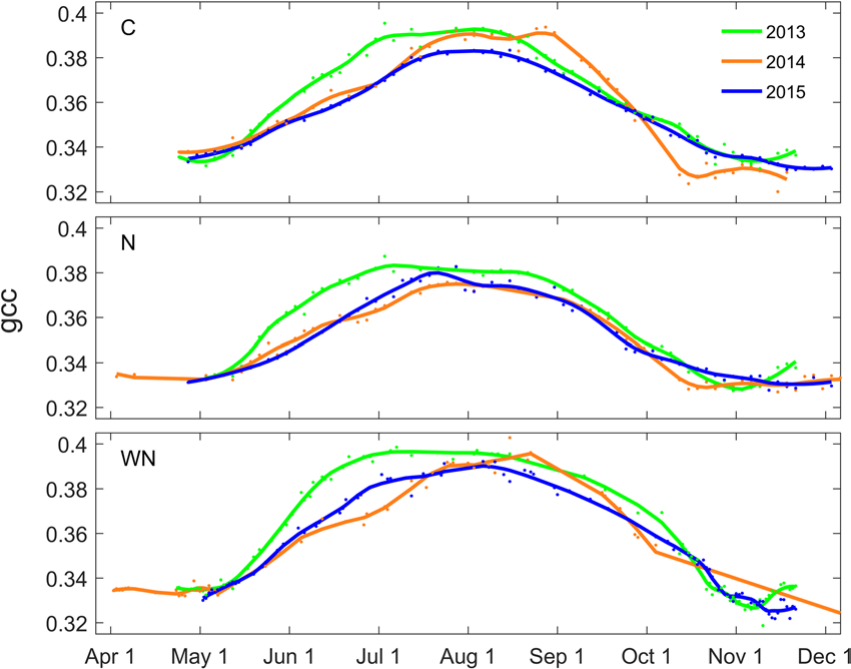
Canopy greenness index (i.e. the green chromatic coordinate, gcc) for control (C), nitrogen (N) and warming + N (WN) plots for 2013–2015. Dots and solid lines represent 3-day means of observed gcc and Loess fits, respectively.

The seasonal development of vascular plant biomass in the C, N and WN plots, as indicated by gcc, started the earliest and was most pronounced during the June-July period in 2013 (Fig. 6), corresponding to similar patterns in GPP_max_. The higher gcc in early 2013 resulted primarily from enhanced graminoid growth (Supplementary Fig. S3). The seasonal and inter-annual variations in gcc were further confirmed by similar temporal patterns in LAI and NDVI (Supplementary Fig. S4) which resulted in strong correlations of gcc with LAI (R^2^ = 0.72 to 0.86) and NDVI (R^2^ = 0.66 to 0.76) (Supplementary Fig. S5). Highest gcc, LAI and NDVI occurred in the WN plot, coinciding with highest vascular plant biomass.

### Seasonal and inter-annual variations in abiotic conditions

**Figure 7:**
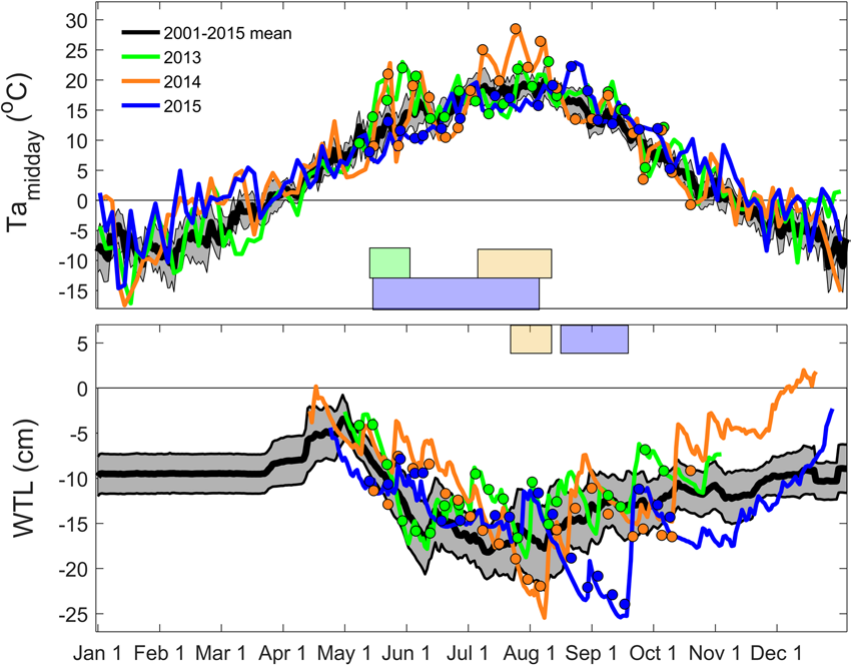
15-year averages (2001–2015) and 2013–2015 midday (10:00 to 14:00) means of air temperature (Ta; top panel) and daily mean water table level below peat surface (WTL; bottom panel) taken from the climate records for the Degerö site. Grey shaded area indicates the 95% confidence interval of the long-term means. The straight line in the 15-year mean WTL data from January to late March indicates frozen conditions during this period. Symbols show midday Ta and manual WTL data collected on the day of field measurements at the experimental plots. Colored boxes indicate the timing of significant abiotic anomalies in Ta and WTL during 2013 (green shade), 2014 (orange shade) and 2015 (blue shade).

To further explore causes for the observed patterns in the seasonal trajectories of GPP_max_, we investigated anomalies in air temperature and water table level dynamics. The results suggest that air temperature and water table level in 2013 were within the range of the 15-year mean (2001–2015) for most of the growing season, except for late spring (May 12 to June 1) which was significantly warmer compared to the 15-year mean and the other two measurement years (*p* = 0.059 in 2014, *p* = 0.008 in 2015) (Fig. 7). This warm spring corresponded closely to the period during which enhanced GPP_max_ and gcc were noted (recall Figs 1 and 6). In 2014, the mid growing season (July 5 to August 8) was significantly warmer and the water table level was significantly lower from July 24 to August 8. This period coincided with the intermittent decrease and peak of GPP_max_ observed in the WN and M plots, respectively, as well as the temporary deviation of the GPP_max_ and gcc trajectories in the C and WN plots (recall Figs 1 and 3). In 2015, the air temperature was significantly lower during most of the growing season (May 13 to August 2) corresponding to the period during which the GPP_max_ trajectory followed most closely that of air temperature. A significant decrease in the water table level from August 15 to September 17 during the same year was noted which, however, had no apparent impact on the GPP_max_ trajectory. Based on the combined air temperature and water table level patterns, we defined the 2013 growing season as ‘meteorologically normal’ due to the absence of strong and persistent deviations in air temperature and water table level. Meanwhile, we consider the 2014 and 2015 growing seasons to have experienced significant constraints from temporarily reduced water table level and lower air temperature, respectively.

## Discussion

The different roles of vegetation types and species-specific contributions with respect to apparent peatland CO_2_ fluxes have been previously highlighted^27,33,54,55^. Here, we build on these previous studies by exploring novel *in-situ* estimates of GPP at the light-saturation level (GPP_max_) across various vegetation types over three meteorologically different growing seasons. Overall, we find that GPP_max_ varied considerably among different vegetation types, over the course of the growing season and among years.

Given the similar abiotic conditions (i.e. in temperature, water and light supply) in the plots during each measurement campaign, the between-plot variation in the GPP_max_ magnitudes were primarily due to differences in the presence of vascular plant and moss species. Additional potential effects from elevated N status enhancing GPP_max_ in the N and WN plots were not apparent. Instead, GPP_max_ was lower in the N and WN plots which was likely due to their reduced moss cover relative to the C plot. This highlights that, despite their lower photosynthetic light saturation level compared to vascular plants (recall Fig. 2), mosses contribute substantially at the ecosystem level not only to apparent GPP e.g.^22^ but also to GPP_max_ in *Sphagnum*-dominated peatland ecosystems.

Seasonal differences in vascular plant and moss GPP_max_ also occurred due to their different responses to abiotic constraints. For instance, the temporary reduction of GPP_max_ in the WN plot during the warm and dry July 2014 suggests a period of water stress for the vascular plant community. In contrast, moss GPP_max_ (in the M plot) increased during the same period, likely as a response to warm temperatures while capillary forces remained sufficient for sustaining water acquisition. In addition, small rainfall events during this period might have further alleviated the drought stress for the moss communities^56,57^. Increased moss GPP_max_ might have compensated for the reduction in vascular plant photosynthesis thereby maintaining community-level GPP_max_ in the C plot at a stable and relatively higher level during this period. This is in line with the classic ecological theory on niche complimentary^58,59^ and indicates that the co-existence and evenness in the biomass ratio of mosses and vascular plants might be crucial for maintaining the carbon sink strength in *Sphagnum*-dominated peatlands during periods with abiotic anomalies. Overall, the contrasting patterns and responses of vascular plant and moss GPP_max_ to biotic and abiotic controls may cause complex dynamics of ecosystem GPP_max_ which must be considered both in flux measurement interpretation as well as in parameterization of process-based models.

Our data further suggest that inter-annual variations of GPP_max_ in the plots containing vascular plants were strongly related to differences in weather patterns and their impact on vascular plant development. For instance, the unusually warm spring in 2013 promoted early green-up and more rapid vegetation development resulting in elevated vascular plant biomass and leaf area. This may explain higher GPP_max_ and earlier timing of peak GPP_max_ in 2013 compared to other years. In contrast, the cooler growing season in 2015 delayed vascular plant development and shifted the timing of peak GPP_max_ in all plots towards early September. Thus, while ecosystem models commonly predict GPP_max_ to reach its maximum during mid-summer^41^, our results suggest a wide intra- and inter-annual range for the timing of its peak. Our findings further highlight that plant phenology represents the integral of effects from preceding abiotic conditions (i.e. in temperature, water and light supply) and thus, phenology is likely to explain GPP_max_ better than each of the individual abiotic variables alone. This also emphasizes the need for an in-depth understanding of phenology responses to abiotic conditions associated with global changes.

Separating the biotic and abiotic controls of carbon fluxes is challenging in peatland ecosystems since information on vegetation phenology and species composition is often compromised by coarse temporal and spatial resolution^38,47^. Many field and modeling studies therefore relate variations in peatland carbon fluxes to mostly abiotic variables e.g.^23,45,60^. Because of the collinearity between plant phenology and abiotic variables (e.g. temperature), these apparent relationships may describe the variations in carbon fluxes reasonably well - except, they do not represent the mechanistic processes driving the peatland carbon cycle and thus may fail to adequately predict its response to disturbance and future global changes.

In this study, we used a canopy greenness index (i.e. gcc) as a continuous proxy for plant development to explore the separate biotic and abiotic controls of GPP_max_. Our main focus was thereby on the seasonal trajectory of GPP_max_ rather than on the daily variation around this trajectory. We demonstrated that the GPP_max_ trajectory followed closely that of gcc during periods without severe abiotic constraints (Fig. 3). This suggests that plant phenology, i.e. biomass development, is commonly the limiting factor and therefore the driver of the GPP_max_ trajectory. This was especially evident during June–July 2013 when the temperature trajectory dropped considerably while the GPP_max_ trajectory continued to follow that of gcc. However, our results also show that abiotic anomalies beyond certain thresholds may cause temporary reductions in GPP_max_ resulting in deviations in its trajectory from that of phenology. Thus, better knowledge of such thresholds and alternating drivers of GPP_max_ during distinct growing season stages is needed to advance our understanding of peatland GPP dynamics.

It is further noteworthy that the sensitivity of the GPP_max_ trajectory to constraints from reduced water availability differed among the various vegetation types. Specifically, the GPP_max_ and gcc trajectories deviated only in the C and WN plots, whereas the GPP_max_ trajectory continued to follow that of gcc in the N plot during the 2014 summer drought period. These contrasting patterns might be due to the lower occurrence of dwarf shrubs in the N plot compared to the C and WN plots (Fig. 4). Since dwarf shrubs maintain a shallow root system within the aerated upper peat layer in comparison to aerenchymatous sedges (e.g. *Eriophorum*), which can extend their roots below the water table level^22,61^, drought impacts on GPP_max_ might be more severe in the presence of a high dwarf shrub cover. This also indicates that findings from our sedge-*Sphagnum* dominated fen might differ for other peatland types such as bogs which are commonly characterized by greater occurrence of shrub species and a lower mean water table level^61–63^.

Global changes including altered climate regimes, more frequent extreme weather events as well as increased atmospheric N deposition and CO_2_ fertilization may severely affect the peatland phenology and carbon cycle^21,55,64–69^. A strength of our study is that changes in vegetation composition did not occur abruptly (i.e. through manual plant removal) but instead resulted from a gradual shift in the environmental conditions achieved through two decades of N addition and artificial warming. This provided a more realistic setting for exploring the impacts of projected global change on vegetation development and its consequences for GPP_max_ dynamics in northern latitudes.

Since GPP_max_ constrains the upper boundary for apparent GPP, better knowledge of the spatial and temporal dynamics of GPP_max_ might help evaluating the consequences of disturbance events and global change on the peatland carbon sink strength. For instance, short-term weather anomalies likely have greater implications for apparent GPP at times when GPP_max_ is high and thereby allowing for greater variability in apparent GPP compared to periods during which low GPP_max_ constrains the potential response range of apparent GPP. Meanwhile, gradual changes in climate and nutrient regimes might trigger a shift in vegetation composition over longer time scales^55,68,70^ which might affect both the magnitude as well as temporal dynamics of GPP_max_. Thus, effects of both short and long-term environmental changes on the apparent GPP will depend on the response range set by the concurrent changes in GPP_max_.

Our study furthermore highlights the value of understanding the separate biotic and abiotic controls which may modulate the responses of GPP_max_ to environmental change during different growing season stages. For instance, limited water availability during a dry mid-summer (July 2014) caused a considerable reduction of GPP_max_. In contrast, a dry spell in late-summer (September 2015) did not reduce GPP_max_ beyond the constraints set by plant phenology. Thus, combining knowledge on the temporal GPP_max_ response characteristics with information on the timing and frequency distributions for projected climate change and extreme weather events e.g.^71,72^ could improve predictions of global change impacts on the peatland carbon cycle.

Based on our results we conclude that spatial and temporal variations in GPP_max_ are primarily driven by species composition and vegetation phenology rather than by concurrent abiotic conditions. Since phenology, however, is itself regulated by preceding abiotic conditions, prolonged anomalies in abiotic conditions (i.e. dry and cold spells) during distinct growing season stages may cause a divergence between the trajectories of GPP_max_ and phenology which may be further modified by species composition. This finding therefore calls for a detailed understanding of phenology responses to global changes. Overall, our study highlights the important role of peatland vegetation ecology in constraining GPP_max_ which has implications for regulating the variations of apparent GPP. It also encourages the use of remotely sensed vegetation indices to estimate ecosystem production. Furthermore, our results emphasize the need for accurate representation of plant functional type- or species-specific vegetation phenology in process-based peatland models as well as for validating model phenology routines (which are driven by meteorological forcings) with field observations, e.g. by the use of phenology cameras.

## Material and Methods

### Site description

The study was conducted at Degerö Stormyr (64°11′N, 19°33′E; 270 m above sea level) which is an oligotrophic, minerogenic, mixed mire system located near the town of Vindeln, county of Västerbotten, Sweden. The 30-year climate reference normals (1961–1990) of annual total precipitation and mean air temperature in the region are 523 mm and +1.2 °C, respectively^73^. The peat depth within the studied area is mostly between 3–4 m. The micro-topography is dominated by irregular mosaic of carpets and lawns (characterized by a mean water table level depth of ~0–10 and ~10–20 cm, respectively), with only few occurrences of hummocks. The vascular plant community within the study area consists mainly of cottongrass (*Eriophorum vaginatum* L.), tufted bulrush (*Trichophorum cespitosum* L. Hartm.), cranberry (*Vaccinium oxycoccos* L.), bog rosemary (*Andromeda polifolia* L.) and cloudberry (*Rubus chamaemorus* L.). *Sphagnum majus* Russ. C. Jens occurs in carpet areas while *S*. *lindbergii* Schimp. and *S*. *balticum* Russ. C. Jens are common in the lawn communities. The hummocks are dominated by *S*. *fuscum* Schimp. Klinggr. and *S*. *rubellum* Wils.^4,63^.

### Experimental design

We made use of a long-term experimental set-up which was established within a representative area of the mire in 1995 (and maintained since then) to explore warming as well as nitrogen and sulfur addition effects on peatland ecology and biogeochemistry^70,74,75^. The experimental plots are surrounded by elevated boardwalks to facilitate measurements while excluding trampling disturbance. From this set-up, we selected one control plot (C), one nitrogen addition plot (N) and one combined warming plus nitrogen addition plot (WN) for this current study and established four replicate measurement locations within each plot. The N and WN plots have received 30 kg N ha^−1^ y^−1^ (with application rates of 10 kg N ha^−1^ in May and 5 kg N ha^−1^ in each following month from June to September) since 1995. In addition, the WN plot was covered with a transparent and punctuated tarp (permeable to water and about 80% of incoming radiation) which raised the mean growing season air temperature inside the plot by 3.6 °C^74^. It is important to note that in this study, we did not intend to investigate the N addition and warming effects *per se*. Instead, these plots were selected for conducting measurements across different vegetation compositions resulting from the treatments over time^70,75^. In 2012, an additional plot was established where vascular plants were repeatedly removed throughout each growing season to obtain a moss-only plot (M).

### Estimates of maximum gross primary production

We estimated maximum gross primary production (GPP_max_) from the difference between net ecosystem exchange (NEE) under full light and ecosystem respiration (ER) measured at four locations within each plot in weekly to bi-weekly intervals between May and October in 2013 to 2015 with the dynamic closed chamber technique^76^. During NEE measurements, a cylindrical chamber (diameter 18.5 cm, height 28 cm) with an opaque sidewall but transparent top was placed onto the collar and an external light-emitting diode (LED) lamp (LED-Light Source SL 3500-C, Photon Systems Instruments, Drásov, Czech Republic) was positioned 5 cm above the chamber. This LED lamp (20 × 20 cm) emitted electrons over the approximate range of photosynthetically active radiation (390–700 nm) with an adjustable photosynthetic photon flux density (PPFD) of 0–2000 µmol m^−2^ s^−1^. We used the radiation level of 2000 µmol m^−2^ s^−1^ for defining non-limiting light conditions since plant photosynthesis commonly saturates between 1500–2000 µmol m^−2^ s^−1 ^^16^. An initial test using a handheld PPFD meter was conducted to ensure that 2000 µmol m^−2^ s^−1^ were obtained at the vegetation surface. The change in chamber headspace CO_2_ concentration was recorded every ~2 seconds over 1–2 minutes by a portable infrared gas analyzer (SBA-4 OEM CO_2_ Analyzer, PP Systems Inc., Amesbury, MA, USA in 2013 and the Ultraportable Greenhouse Gas Analyzer, Los Gatos Research Inc., Mountain View, CA, USA in 2014 and 2015) connected in a closed loop to the chamber. Fluxes were computed from the linear concentration change over time corrected for air density using the ideal gas law. More details on the flux measurement procedure are provided in the Supplementary Information S1 as well as by Vermeij^77^ and de Goede^78^.

### Estimates of GPP-light response function parameters

In 2013, we determined *in-situ* GPP-light response curves for all plots in monthly intervals from May to September based on repeated measurements (in ~5 min intervals) at light levels of 2000, 1500, 1000, 500 and 0 µmol m^−2^ s^−1^ supplied by the LED lamp described above. In contrast to light response curves derived from data collected under various ambient light conditions over several weeks, these light response curves were obtained within <1 hour and thus exclude confounding effects from concurrent changes in biotic and abiotic conditions. We estimated the parameters α (the initial slope of the light response curve; unitless) and A_max_ (the maximum assimilation at light saturation; µmol CO_2_ m^−2^ s^−1^) from the hyperbolic relationship between GPP and PPFD (Eq. 2).

2 $$GPP=\,\frac{\alpha \times PPFD\times {A}_{{\max }}}{\alpha \times PPFD+{A}_{{\max }}}$$

### Vegetation biomass and phenology

#### Biomass and leaf area index

We determined vascular plant aboveground biomass and leaf area index (LAI) in weekly to bi-weekly intervals in ten micro-plots (7.5 × 7.5 cm) within each of the C, N and WN plots (see^47^ and Supplementary Information S2 for details on the inventory measurement procedure). Moss cover was estimated for each plot in 2013 by visually assessing the percentage of area covered by moss. Assuming similar shoot density at all plots, photosynthetically active moss biomass was derived from multiplying moss cover with capitulum biomass previously determined for nearby undisturbed areas (i.e. similar to the C plot) at the same site^63^.

#### Canopy greenness index

To track vascular plant development with high temporal resolution and spatial representation of the entire experimental plot, we derived a canopy greenness index from hourly images obtained by digital repeat photography at the C, N and WN plots. Digital point-and-shoot cameras (A480, Canon, Tokyo, Japan) were installed next to each plot at 1.2 m height with an off-nadir angle of 57.5 degrees (Supplementary Fig. S1). A camera model specific intervalometer script (Canon Hack Development Kit; http://chdk.wikia.com) was used to collect images at hourly intervals. The white balance was fixed to ‘daylight’ (resulting in a white balance temperature of 5200 Kelvin) to ensure a consistent and neutral color profile. As the tarp covering the WN plot was not fully transparent, it was temporarily removed at 3-day to weekly intervals and images at the WN plot were taken on those days within midday hours (10:00 to 14:00). We calculated a canopy greenness index based on the green chromatic coordinate (gcc) for each image as:

3 $$gcc=\frac{G}{R+G+B}$$

where R, G and B are the digital numbers (0–255) of the red, green and blue image channels within a selected region of interest (shown in Supplementary Fig. S1) as described in Peichl *et al*.^48^ and Sonnentag *et al*.^79^.

#### Normalized difference vegetation index

To obtain a proxy for the seasonal changes in photosynthetic capacity related to the leaf chlorophyll content^80,81^, we determined the normalized difference vegetation index (NDVI) from reflectance measurements at red (660 nm) and near infrared (840 nm) wavelengths using the FieldScout CM 1000 NDVI Chlorophyll Meter (Spectrum Technologies, Inc., Aurora, IL, USA). In each of the C, N and WN plots, measurements were taken over a grid of 30 sampling points in weekly to bi-weekly intervals.

### Abiotic variables

Ambient air temperature, atmospheric air pressure and precipitation were continuously recorded at a meteorological station located approximately 100 m away^35^. Water table level below the peat surface was measured within the experimental area using a float and counterweight system attached to a potentiometer^82^. Overall, the proximity of our experimental plots and the controlled light supply from the LED lamp ensured that abiotic conditions were similar during each measurement campaign and followed the same seasonal pattern in all plots. This experimental set-up therefore enabled us to explore how vegetation composition and phenology affected between-plot differences in GPP_max_.

### Seasonal trajectories of GPP_max_, gcc, air temperature and water table level

We explored the drivers of the temporal changes in GPP_max_ by deriving the seasonal trajectories of GPP_max_ and of its main biotic and abiotic controls, i.e. gcc, air temperature and water table level, for the C, N and WN plots. We used here gcc as proxy for plant development as it provided a continuous (i.e. daily) and spatially more representative (by integrating over the entire plot) estimate compared to LAI or NDVI whose measurement footprints were constrained to small sampling areas (few tens of cm^2^) and weekly to bi-weekly temporal resolution. We first normalized each data set relative to the year 2013 which was considered as ‘meteorologically normal’ (see Results section on abiotic conditions). We then applied a Loess smoothing curve fit through the measured data points as an estimate of the seasonal trajectories for GPP_max_, gcc, air temperature and water table level.

### Statistical analysis

To avoid pseudo-replication when assessing between-plot differences, flux and vegetation data from the various sampling locations within each plot were averaged and further statistical analysis was conducted on the plot means^83^. Between-plot differences in mean GPP_max_ and vascular plant biomass as well as between-year differences in air temperature and water table level during specific periods were assessed with the non-parametric Friedman one-way analysis of variance by ranks test for repeated (i.e. dependent) measurements followed by a Bonferroni post-hoc comparison. The significance level (*p*) was 0.05 unless stated otherwise. To explore which of the biotic and abiotic variables best explained the seasonal GPP_max_ trajectory, the ‘similarity’ between the trajectory of GPP_max_ and those of gcc, air temperature and water table level was assessed by comparing the cumulative deviation (I_dev_) between the trajectories (Supplementary Fig. S6). Uncertainty estimates for the GPP_max_ trajectories and I_dev_ were derived from the variation among the trajectories of the individual flux collars (*n* = 4) in each plot. The first rule or error propagation was used to obtain the cumulative uncertainty in I_dev_. All data analysis was conducted using the Matlab software (Matlab R2015a, Mathworks, USA).

### Data availability

The datasets generated during and/or analysed during the current study are available from the corresponding author on reasonable request.

## Electronic supplementary material

Supplementary Information
