# Supplementary material for: Peatland vegetation composition and phenology drive the seasonal trajectory of maximum gross primary production
Source: Sci Rep. 2018 May 22;8:8012. doi: 10.1038/s41598-018-26147-4 (PMC5964230; doi:10.1038/s41598-018-26147-4)
Supplement: Supplementary file 1 — Supplementary Information [file 41598_2018_26147_MOESM1_ESM.docx]

**Peatland vegetation composition and phenology drive the seasonal trajectory of maximum gross primary production**

Matthias Peichl^1*^, Michal Gažovič^1^, Ilse Vermeij^2^, Eefje de Goede^3,4^, Oliver Sonnentag^5^, Juul Limpens^2^, Mats B. Nilsson^1^

^1^ Department of Forest Ecology and Management, Swedish University of Agricultural Sciences, 90183 Umeå, Sweden

^2^ Plant Ecology and Nature Conservation Group, Wageningen University, 6708 PB Wageningen, the Netherlands

^3^ Department of Aquatic Ecology, Radboud University Nijmegen, 6525 AJ Nijmegen, the Netherlands

^4^ Institute of Environmental Sciences, Leiden University, 2333CC Leiden, the Netherlands

^5^ Département de géographie, Université de Montréal, Montréal, QC H2V 2B8, Canada

**^*^**Author for correspondence:

Email: matthias.peichl@slu.se

**Supplementary Information S1: Description of chamber flux measurements**

In 2012, we inserted four replicate collars (diameter 18.5 cm) approximately 5 cm deep into the peat in each of the experimental plots. At each collar, we first measured NEE under full light conditions (i.e. 2000 µmol photons m^-2^ s^-1^). For this purpose, the lamp was placed above the collar at least 1 minute prior to chamber deployment to allow the vegetation to adjust to the full light conditions. An initial test showed that the LED lamp did not increase the chamber air temperature. Following the NEE measurement, the chamber was removed, vented and placed back onto the collar with the top darkened by a heavy-duty plastic foil. A second measurement was then taken under dark conditions to estimate ER. The tarp covering the WN plot was removed during the measurements. During each measurement, the CO_2_ concentration was recorded in ~2 second intervals over 1-2 minutes by a portable infrared gas analyzer connected in a closed loop to the chamber. The return sample air flow (~1 l/min) ensured continuous mixing of the headspace air. In 2013, a custom-made analyzer containing a measurement cell from PP Systems (SBA-4 OEM CO_2_ Analyzer, PP Systems Inc., Amesbury, MA, USA) was used while a gas analyzer from Los Gatos (Ultraportable Greenhouse Gas Analyzer, Los Gatos Research Inc., Mountain View, CA, USA) was used in 2014 and 2015. A recent inter-comparison of different gas analyzers suggests that gas analyzer make and model has no significant impact on the measured fluxes when using the same chamber set-up (unpublished data). Fluxes were computed from the linear concentration change over time corrected for air density using the ideal gas law. The gas analyzers were connected to handheld computers which displayed the concentration changes in real-time. Any measurement deviating from a steady linear increase could thus be identified, discarded and repeated on-site which resulted in all fluxes having a coefficient of determination (R^2^) > 0.80. Except, we kept low fluxes (i.e. close to zero) which naturally have a lower R^2^. The threshold values for defining low fluxes were determined based on a regression between fluxes and R^2^ suggesting <0.05 and <|0.1| µmol CO_2_ m^-2^ s^-1^ for ER and NEE, respectively.

**Supplementary Information S2: Estimates of vascular biomass and leaf area index**

Vascular plant aboveground biomass and leaf area index (LAI) were determined in weekly to bi-weekly intervals in ten micro-plots (7.5x7.5 cm) within each of the C, N and WN plots following Wilson et al. ^1^. In each micro-plot, the number of leaves and the average leaf length (for herbs) or leaf size (for woody shrubs) was recorded separately for every species. Once a month, 10-20 representative leaves (covering different leaf lengths and sizes) were collected per species outside of the permanent micro-plots. These samples were scanned, oven-dried and weighted to develop leaf length/size-biomass and length/size-area relationships (R^2^ = 0.85 to 0.99) which were then used to upscale biomass and leaf area to the experimental plot area.

1. Wilson, D. *et al.* A high resolution green area index for modelling the seasonal dynamics of CO2 exchange in peatland vascular plant communities. *Plant Ecol* **190,** 37–51 (2007).

**Supplementary Figures**

**S1:**


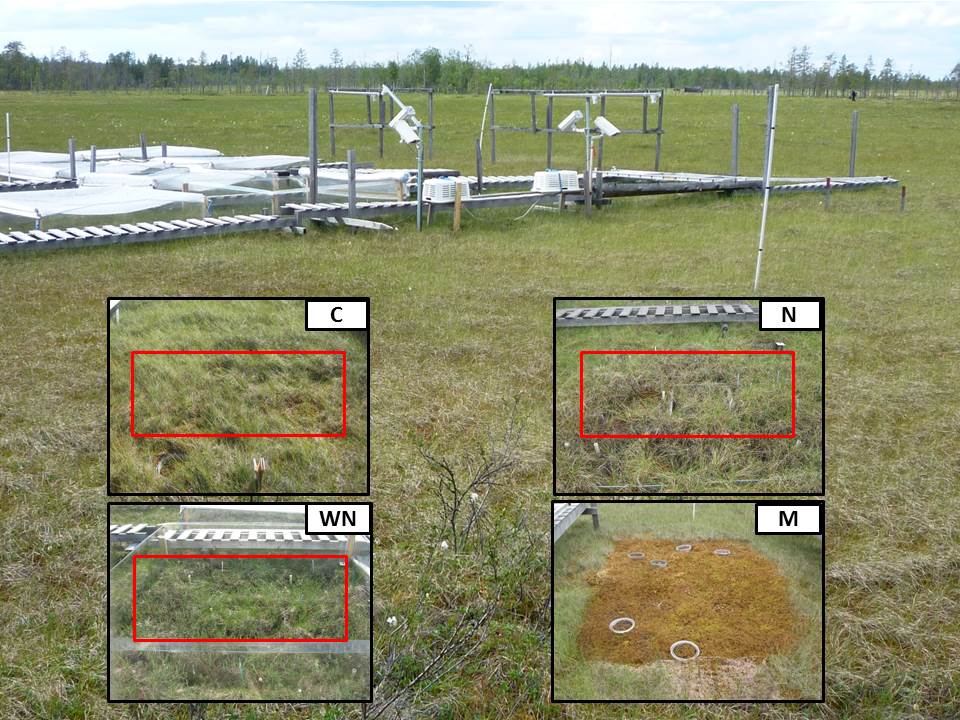


**Figure S1:** Image of the experimental set up at the Degerö peatland with detailed view on control (C), nitrogen (N), warming + N (WN) and moss (M) plots; red frames indicate the region of interest used in the image analysis of the phenology cameras to obtain a canopy greenness index (gcc).

**S2:**

**Figure S2:** Boxplots for maximum gross primary production (GPP_max_; June to August) measured under full light conditions (2000 µmol photons m^-2^ s^-1^) in control (C), nitrogen (N), warming + N (WN) and moss (M) plots during the growing seasons of 2013-2015. Different letters indicate significant differences (*p* < 0.05).

**S3:**

**Figure S3:** Time series for graminoid and shrub aboveground (green) biomass in control (C), nitrogen (N), and warming + N (WN) plots based on manual vegetation inventory over the growing seasons 2013-2015.

**S4:**

**Figure S4:** Vascular plant leaf area index (LAI) and normalized difference vegetation index (NDVI) for control (C), nitrogen (N) and warming + N (WN) plots for 2013-2015.

**S5:**


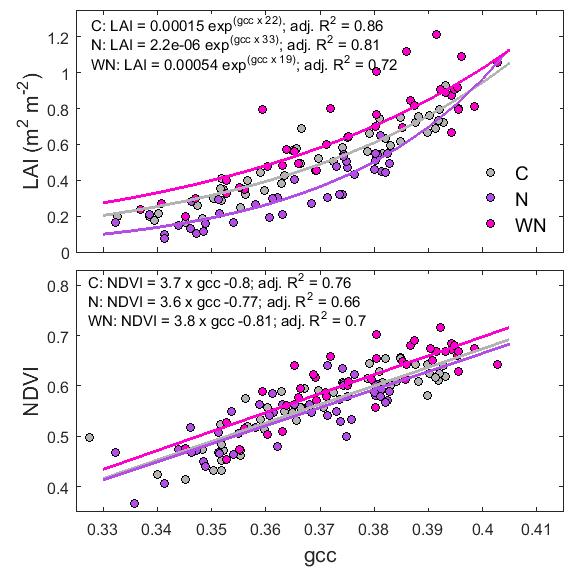


**Figure S5:** Regression relationship between the canopy greenness index based on the green chromatic coordinate (gcc) and leaf area index (LAI) (top panel) and between gcc and normalized difference vegetation index (NDVI) (bottom panel) in control (C), nitrogen (N), and warming + N (WN) plots bulked over the growing seasons 2013-2015. Lines show exponential fits (top panel) and linear fits (bottom panel), respectively.

**S6:**

**
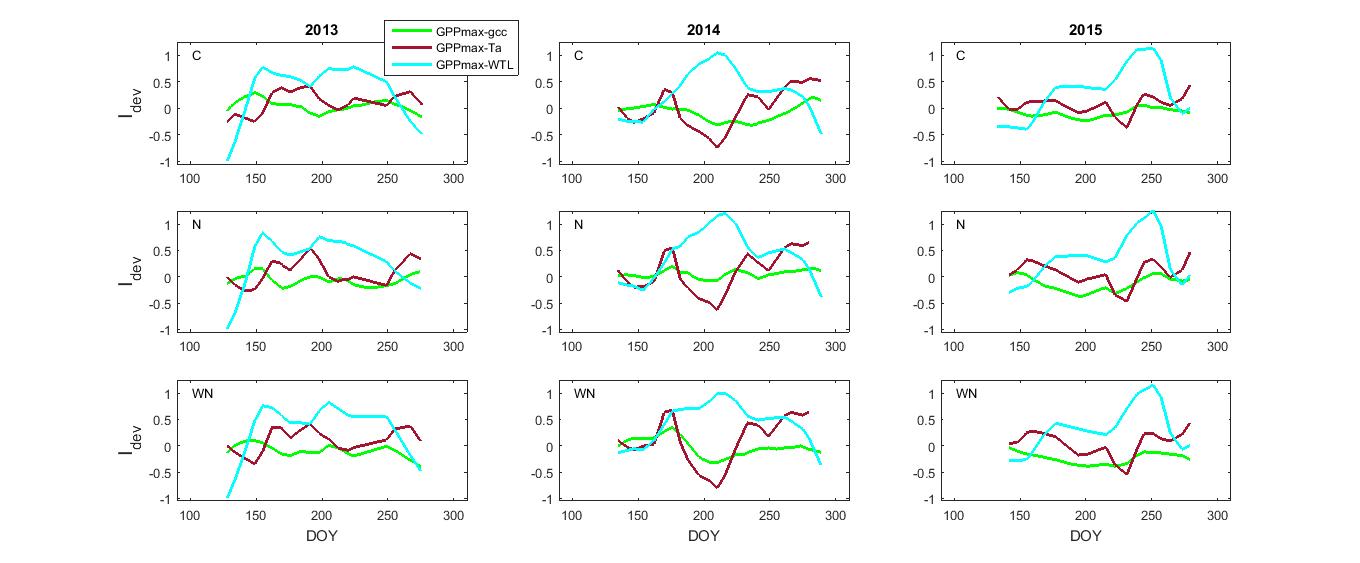
**

**Figure S6:** Index of deviation (I_dev_) indicating the departure between the normalized trajectories of maximum gross primary production (GPP_max_) and the normalized trajectories of the green chromatic coordinate (gcc), air temperature (Ta) and water table level (WTL) in control (C), nitrogen (N), and warming + N (WN) plots during the growing seasons of 2013-2015.
